# Editorial Comment from Dr Kojima to Possible abscopal effect after discontinuation of nivolumab in metastatic renal cell carcinoma

**DOI:** 10.1002/iju5.12203

**Published:** 2020-07-24

**Authors:** Takahiro Kojima

**Affiliations:** ^1^ Department of Urology Faculty of Medicine University of Tsukuba Tsukuba Japan

The abscopal effect is a rare phenomenon that describes the regression of distant lesions outside the treatment field of radiation therapy (RT). So far, this rare effect has been reported for several cancers which are considered to be immunogenic, including renal cell carcinoma (RCC).^1^ The abscopal effect has been considered to be an immune‐mediated phenomenon, suggesting that combination of immunotherapy and RT could potentially synergize.

The article by Nakajima *et al*. reported that the patient with advanced RCC who showed the tumor reduction in non‐irradiated metastases at 9 months after nivolumab discontinuation.^2^ There are only a few case series describing the abscopal effect by sequential treatment with nivolumab and RT in patients with advanced RCC. This report indicates that RT and anti‐PD1 antibody combination therapy is a possible treatment option for metastatic RCC. However, there are many practical unanswered questions regarding the combination of RT and immunotherapy. Who can receive treatment benefit? What is the optimal sequencing of immunotherapy and RT? What is the optimal dose and fraction of RT? What is the optimal site for RT? Several ongoing trials which are evaluating the combination of RT with immunotherapy in patients with RCC will reveal these questions and the real meaning of combination therapy.^3^ Nevertheless, anecdotal clinical evidences are still important and valuable resources that may lead to new research and advances in clinical practice.

## Conflict of interest

The author declares no conflict of interest.
